# Network pharmacology-based insights into the role of gut microbiota metabolites in insulin resistance

**DOI:** 10.3389/fmicb.2025.1617496

**Published:** 2025-06-23

**Authors:** Bing Xiao, Xin Chen, Ruiyu Zong, Yiming Guan, Zhu Zhu, Siling Bi

**Affiliations:** ^1^College of Acupuncture and Massage, Shandong University of Traditional Chinese Medicine, Jinan, China; ^2^College of Health Sciences, Shandong University of Traditional Chinese Medicine, Jinan, China; ^3^Medical College, Shandong University of Traditional Chinese Medicine, Jinan, China

**Keywords:** network pharmacology, gut microbiota, gut microbiota metabolites, insulin resistance, molecular mechanism

## Abstract

**Background:**

Extensive research has demonstrated that the gut microbiota plays a critical role in maintaining homeostasis and promoting overall human health. However, the pharmacological mechanisms and functional roles of gut microbiota metabolites remain insufficiently understood. This study employs a network pharmacology approach to elucidate the metabolic transformation processes of gut microbiota metabolites and their molecular mechanisms in the pathogenesis of insulin resistance (IR), aiming to uncover the complex interactions among gut microbiota, metabolites, and therapeutic targets.

**Methods:**

Gut microbiota metabolites and their corresponding target genes were retrieved from the gutMGene database. Potential targets of the metabolites were predicted using the SEA and STP databases. Disease-related targets for insulin resistance were collected from the GeneCards, DisGeNET, and OMIM databases. Core targets were identified via a protein–protein interaction (PPI) network, followed by comprehensive GO and KEGG enrichment analyses. Finally, a network illustrating the relationship among microbiota-substrate-metabolite-target was established.

**Results:**

Thirteen overlapping targets between the gut microbiota and insulin resistance were identified, among which IL6, JUN, and PPARG were recognized as hub genes. The MSMT (microbiota-substrate-metabolite-target) network revealed that these three hub genes exert therapeutic effects through 10 gut microbiota metabolites, 10 substrates, and 21 microbial species. KEGG pathway analysis indicated that the IL-17, Toll-like receptor, HIF-1, NOD-like receptor, TNF, and VEGF signaling pathways are the primary pathways involved in the pathogenesis of IR.

**Conclusion:**

Gut microbiota metabolites may exert therapeutic effects on insulin resistance primarily through the targets IL6, JUN, and PPARG. The regulatory mechanisms are likely associated with several key signaling pathways, including the IL-17, Toll-like receptor and HIF-1, pathways. These three pathways collectively form an interconnected inflammation-metabolism-hypoxia network. Targeting key nodes within this network—such as the IL-17 receptor, TLR4, or HIF-1α—may offer a multidimensional therapeutic strategy for insulin resistance (IR) and its associated complications.

## Introduction

Insulin resistance (IR), long overlooked as an independent risk factor, currently affects approximately 51% of the global population, with its prevalence continuing to rise in both developed and developing countries (Fazio et al., 2024). IR plays a central role in the development of non-communicable diseases such as type 2 diabetes and cardiovascular disorders (Goh et al., 2022). Epidemiological investigations of IR are essential for assessing disease burden and guiding evidence-based public health interventions and clinical decision-making. However, systematic screening and preventive strategies for IR remain lacking in the general population (Chiefari et al., 2021). As a result, IR has been referred to as a “silent epidemic” and has emerged as one of the most critical public health issues of the 21st century (Hernández-Valdez et al., 2023).

The etiology of IR is multifactorial, with obesity recognized as a major modifiable risk factor. Additionally, IR may result from medication use (e.g., glucocorticoids, antiretroviral drugs, and oral contraceptives), disorders of lipid metabolism, genetic defects in insulin signaling (type A IR), or autoimmune responses involving insulin receptor-blocking antibodies (type B IR) (Chockalingam et al., 2021).

IR is a key pathogenic component in a variety of metabolic diseases, including type 2 diabetes, and is defined as a reduced responsiveness of insulin-targeted tissues to physiological insulin levels (Armutcu and McCloskey, 2024). It is characterized by impaired glucose uptake and metabolism in skeletal muscle and adipose tissue, inadequate suppression of hepatic gluconeogenesis, and uncontrolled lipolysis in adipose tissues (Rivas and Nugent, 2021). Consequently, IR contributes to the development of type 2 diabetes (Elkanawati et al., 2024), metabolic syndrome (Tahapary et al., 2022), and ischemic stroke, and is closely associated with poor prognosis (Fan et al., 2024). Numerous studies have also linked IR to alterations in cardiovascular structure and function, such as myocardial hypertrophy and ventricular remodeling, which are key features in the pathogenesis of diabetic heart disease (DHD) (Li et al., 2020). These findings underscore the urgent need to investigate the pathophysiological mechanisms underlying IR and to develop effective therapeutic strategies.

The gut microbiota (GM), often considered an overlooked organ system, has garnered increasing attention in recent years (Ago et al., 2018). The human gastrointestinal tract harbors trillions of microorganisms, including bacteria, fungi, and viruses, with bacteria constituting the majority (Ahlawat et al., 2021). The gut microbiota is diverse yet relatively stable, with a shared core microbiome dominated by two major phyla: *Bacteroidetes* and *Firmicutes* (Zhou et al., 2020). The microbial community comprises over 114 bacterial species and weighs approximately 1–2 kg, earning it the designation of the “second genome” of the human body (Fassarella et al., 2021). The gut microbiota plays a vital role in maintaining human health through its involvement in nutrient absorption, energy metabolism, immune regulation, and maintenance of the intestinal barrier (Liu et al., 2023; Qiu et al., 2022). Beneficial gut bacteria can also exert immunosuppressive effects by modulating host immune responses (Fan and Pedersen, 2021).

Dysbiosis of the gut microbiota has been implicated in the pathogenesis of various diseases beyond the gastrointestinal tract, including metabolic disorders, cardiovascular diseases, and neurological conditions (Yang et al., 2023). For instance, gut microbiota composition is closely linked to hypertension (Wang et al., 2022), with pathogenesis involving complex interactions along the metabolite-immune axis and the microbiota-gut-brain axis. Increasing evidence suggests that this axis also contributes to the regulation of sleep behavior and may play a pivotal role in sleep disorders (Isticato, 2023).

Moreover, specific microbial taxa, such as Bifidobacterium and Akkermansia, have been positively correlated with improved insulin sensitivity. Akkermansia strengthens the intestinal mucus layer and upregulates mucin gene expression, thereby reducing the translocation of lipopolysaccharide (LPS) into the circulation—a process known to exacerbate IR and diabetes-related complications (Li et al., 2023). In obese individuals, alterations in gut microbial composition are closely linked to IR, with Bifidobacterium abundance often inversely associated with IR severity (Aljuraiban et al., 2023). Additionally, the gut microbiota can influence host metabolism by modulating bile acid metabolism, increasing levels of lithocholic acid (LCA), and producing succinate, further contributing to the amelioration of IR. These findings suggest that gut microbes, through diverse mechanisms, profoundly influence host metabolic homeostasis and play an essential role in improving IR.

**Figure 1:**
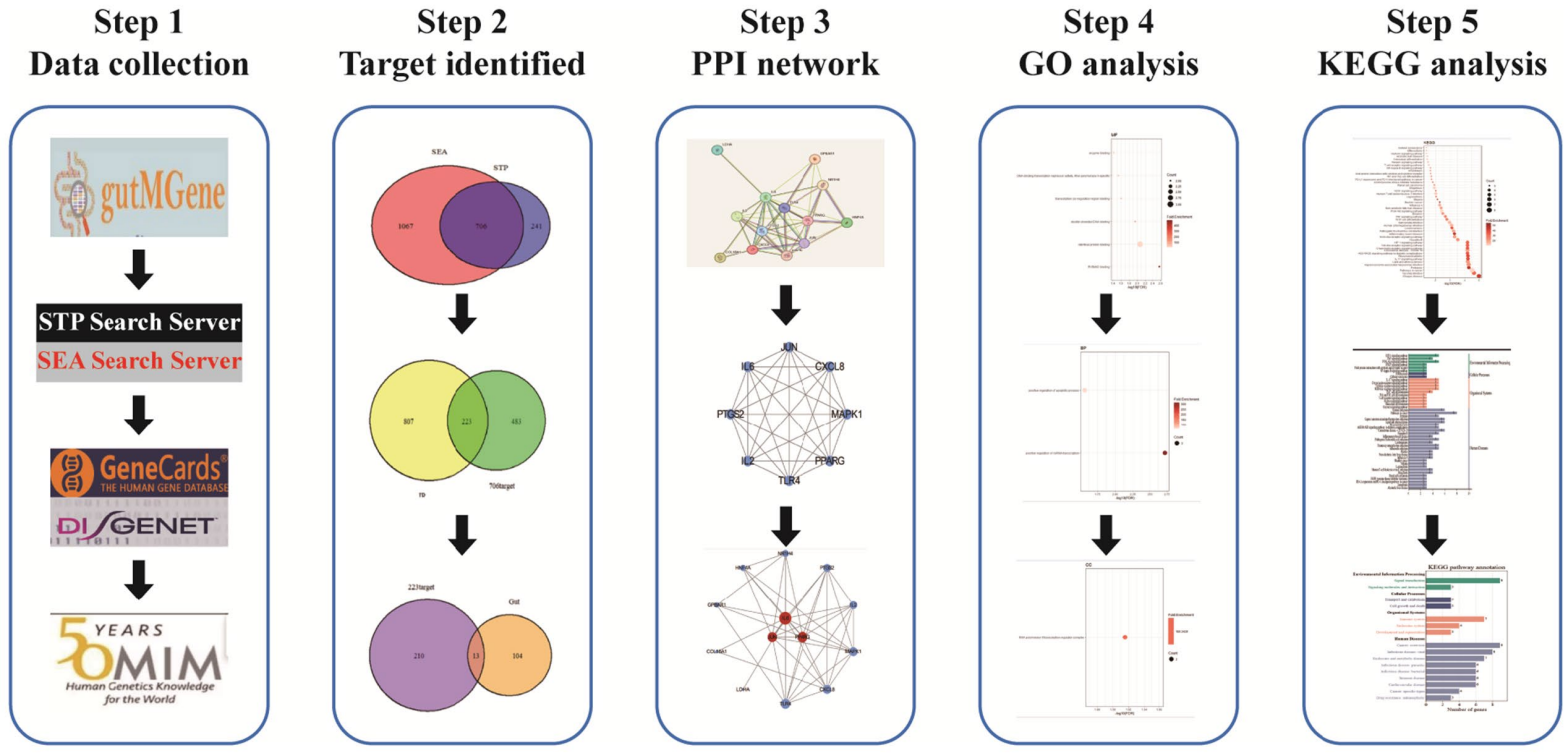
The flowchart reflecting our study design.

In recent years, therapeutic strategies targeting gut microbiota modulation have demonstrated promising efficacy and relatively few side effects, positioning the microbiota as a potential intervention point for IR management. However, the roles and mechanisms of gut microbiota-derived metabolites in the context of IR remain insufficiently explored. Network pharmacology, which integrates drug-target-disease interactions within biological networks, offers a valuable framework for unraveling the molecular mechanisms of complex diseases. This approach facilitates the rapid identification of key bioactive components and therapeutic targets. In the present study, we applied network pharmacology to systematically investigate the metabolic transformation of gut microbiota-derived compounds and to elucidate the molecular mechanisms by which they influence the development of IR. The overall research workflow is illustrated in Figure 1.

## Materials and methods

### Identification of metabolites and targets of gut microbiota

We acquired gut microbiota metabolites and gut-associated target genes from the gutMGene v2.0 database.^1^ The metabolites were then converted into SMILES (Simplified Molecular Input Line Entry System) format using the PubChem database.^2^ Subsequently, potential targets of the metabolites could be predicted by using the SEA and STP databases, with the species restricted to *Homo sapiens*. The overlapping targets predicted by both methods were identified using a Venn diagram and selected as the final set of candidate targets.

### Identification of disease targets

Using “Insulin Resistance” as keyword, we search for Insulin Resistance-related disease targets through GeneCards,^3^ DisGeNET,^4^ and OMIM^5^ databases. From the GeneCards database, only targets with a relevance score ≥10 were included for further analysis. Overlapping disease targets across the three databases were identified using a Venn diagram and defined as the final set of IR-related targets.

### PPI network construction and analysis

The final targets of IR were overlapped with the targets of gut microbiota metabolites using a Venn diagram to identify common targets. These overlapping targets were then submitted to the STRING database,^6^ with the interaction score threshold set to a combined score >0.4, to construct a protein–protein interaction (PPI) network. The resulting PPI network was subsequently visualized, and hub genes were identified based on network topology analysis.

### GO and KEGG enrichment analysis

‌The overlapping targets were uploaded to the DAVID database^7^ for GO and KEGG enrichment analyses. The *p*-values of the enriched terms were adjusted using the Benjamini–Hochberg method, and terms with adjusted *p*-values <0.05 were retained for further analysis. A false discovery rate (FDR) threshold of <0.05 was then applied to identify significantly enriched GO and KEGG terms. Additionally, a minimum gene count threshold of ≥5 was set to filter out low-abundance annotations. The final enrichment results were visualized using a bioinformatics platform.

### The evaluation of and toxicity

SwissADME^8^ and ADMETlab^9^ platforms were used to evaluate the pharmacokinetic and toxicity profiles of key metabolites. A total of 32 metabolites associated with the three hub genes were assessed for drug-likeness and toxicity. High-potential candidate drug molecules were identified based on Lipinski’s rule of five, including criteria such as molecular weight (MW) <500 Da, hydrogen bond donors (HBD) <5, Moriguchi LogP (MlogP) <5, and polar surface area (PSA) <140, to ensure their suitability for clinical application.

## Results

### The identifications of targets of gut microbiota metabolites intervening IR

**Figure 2:**
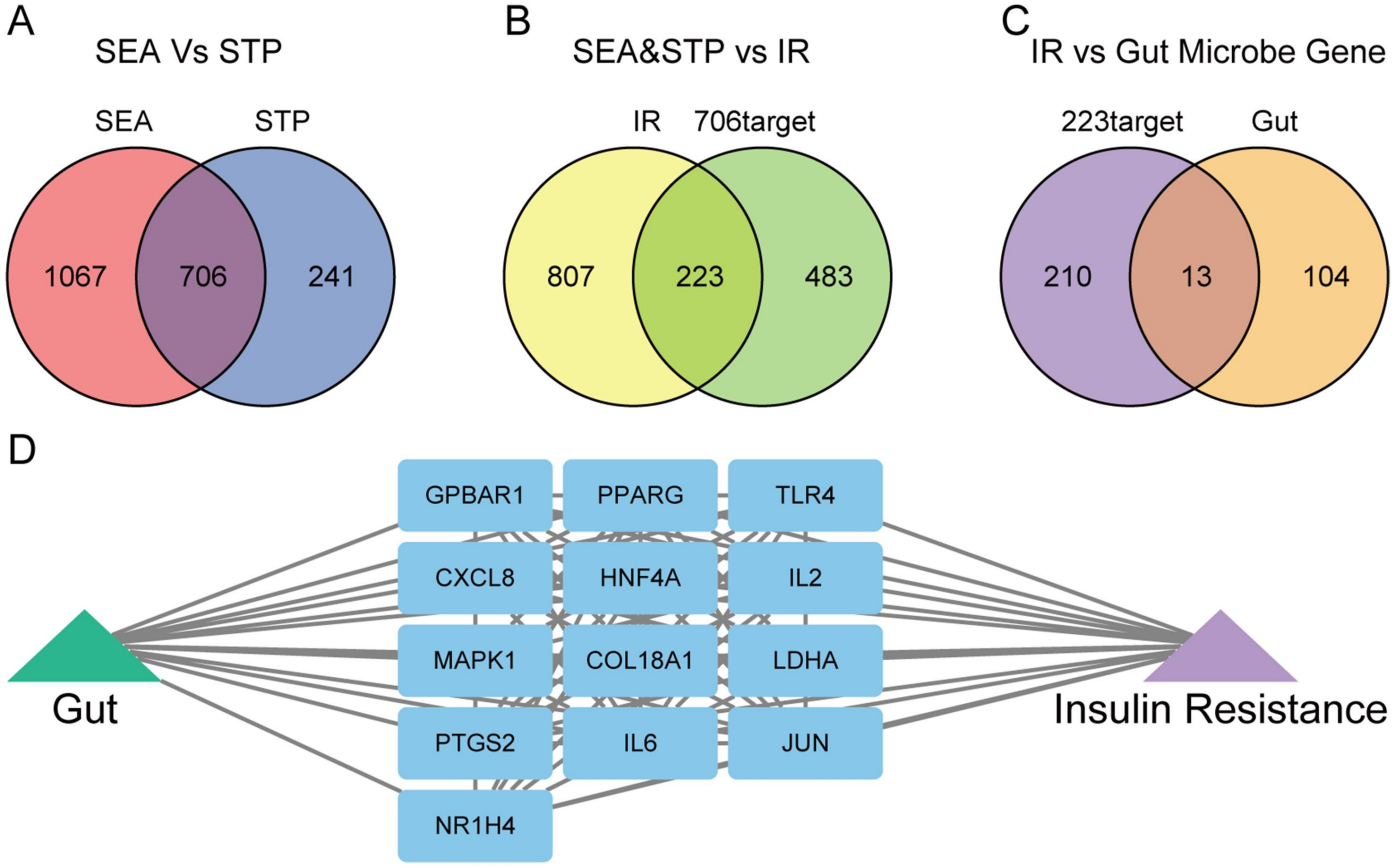
Identification of gut microbiota metabolites and IR-related targets. **(A)** Common targets of gut microbiota metabolites predicted by the SEA and STP databases. **(B)** Common targets between gut microbiota metabolites and insulin resistance (IR). **(C)** Common targets between gut microbiota metabolites-IR and human gut targets. **(D)** The network of gut-targets-IR.

A total of 251 gut microbiota metabolites, 228 metabolites with available structural information (in SMILES format), and 117 gut-associated target genes were retrieved from the gutMGene v2.0 database. Using the SEA and STP platforms, 1,773 and 947 targets were predicted for the 251 metabolites, respectively. A Venn diagram revealed 706 overlapping targets, which were identified as the final set of potential targets for the gut microbiota metabolites (Figure 2A).

From the GeneCards, DisGeNET, and OMIM databases, 1,030 insulin resistance (IR)-related targets were identified. By overlapping these with the 706 gut microbiota metabolite targets, 223 common targets were obtained (Figure 2B). Further intersection of these 223 targets with gut-associated genes yielded 13 final overlapping targets (Figure 2C). A network illustrating the interactions among gut microbiota, targets, and IR was then constructed to visualize the regulatory relationships (Figure 2D).

### PPI network construction and analysis

**Figure 3:**
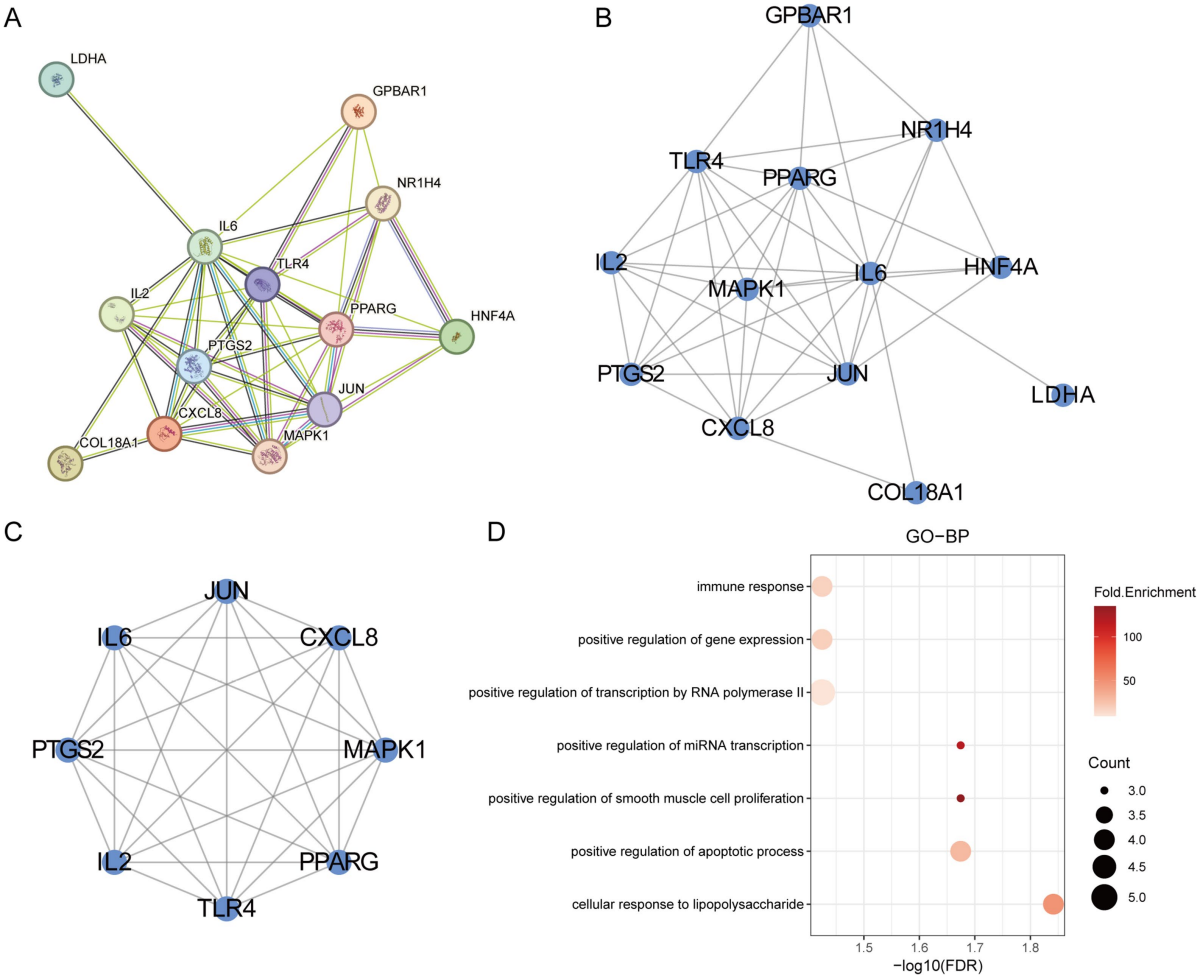
PPI network analysis. **(A)** The PPI network of the overlapping targets. **(B)** The visualization of the PPI network. **(C)** The cluster of PPI. **(D)** The GO-BP analysis of cluster.

The 13 final targets were uploaded to the STRING database to generate a protein–protein interaction (PPI) network (Figure 3A). Cytoscape software was used for visualization, revealing 13 nodes and 44 edges in the network (Figure 3B). Based on degree centrality (DC) values, the top three hub genes—IL6, JUN, and PPARG—were identified as the core targets modulated by gut microbiota metabolites. The cluster of the PPI network was identified a functional module containing 8 targets and 28 edges (Figure 3C). GO biological process (GO-BP) enrichment analysis of the cluster indicated enrichment in pathways such as immune response, cellular response to lipopolysaccharide, and positive regulation of apoptotic process (Figure 3D). These results suggest that the PPI network may contribute to the pathogenesis of IR through the regulation of immune responses.

### GO enrichment analysis

**Figure 4:**
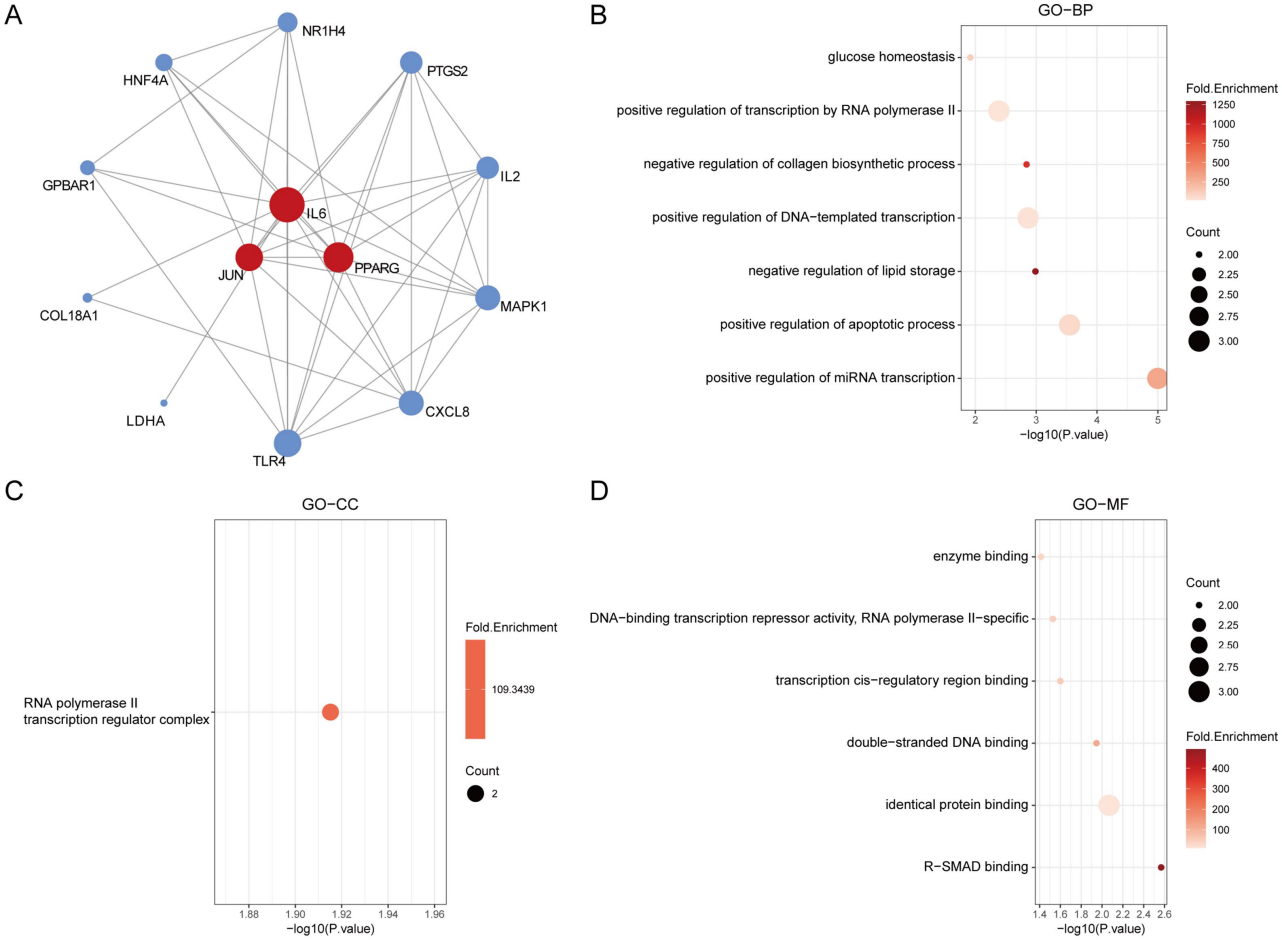
The GO enrichment analysis of core IR targets regulated by gut microbiota metabolites. **(A)** The hub genes of PPI network. **(B)** The GO-BP analysis of hub genes. **(C)** The GO-CC analysis of hub genes. **(D)** The GO-MF analysis of hub genes.

Gene Ontology (GO) enrichment analysis is a bioinformatics approach used to explore the functional enrichment of a gene set within the GO categories. First, we visualized the hub genes involved in the regulation of insulin resistance (IR) (Figure 4A). GO enrichment analysis of the 13 final targets revealed that, in terms of biological processes (GO-BP), gut microbiota metabolites may regulate IR through positive regulation of miRNA transcription, apoptotic process, and DNA-templated transcription and transcription by RNA polymerase II (Figure 4B). The associated cellular component (GO-CC) was primarily the RNA polymerase II transcription regulator complex (Figure 4C). The TOP1 MF (molecular function) is closely related to identical protein binding (Figure 4D). These findings suggest that the hub genes may influence the development of IR by modulating miRNA transcription and apoptotic pathways.

### KEGG enrichment analysis

**Figure 5:**
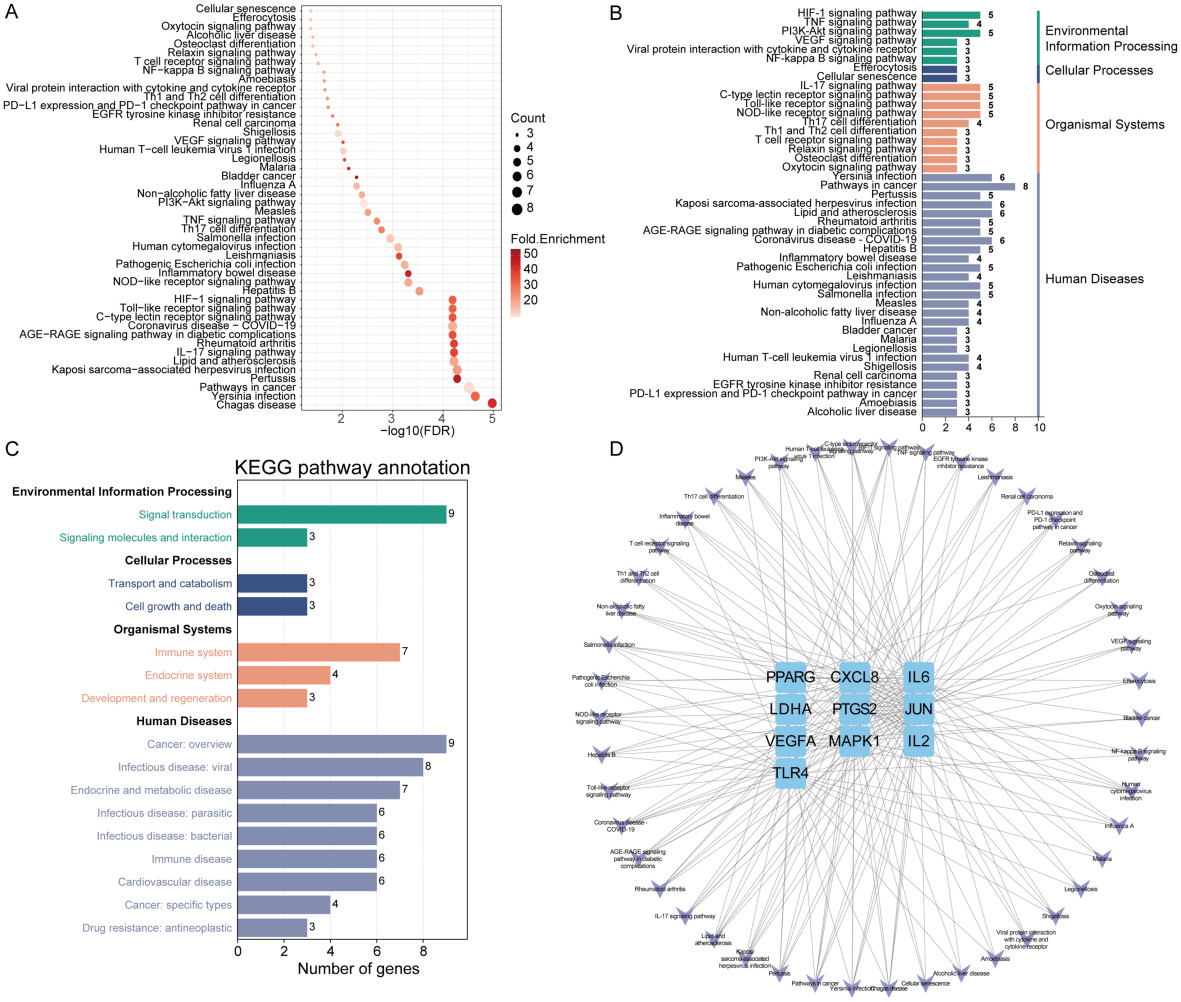
The KEGG enrichment analysis of core IR targets regulated by gut microbiota metabolites. **(A)** KEGG pathway analysis. **(B)** KEGG classification analysis. **(C)** KEGG pathway annotation analysis. **(D)** The network of targets-pathways. The V shape represents pathway, the blue rectangle represents target.

KEGG enrichment analysis was performed to further elucidate the potential biological pathways. Results indicated that gut microbiota metabolites may exert regulatory effects on IR via the IL-17 signaling pathway, Toll-like receptor signaling pathway, HIF-1 signaling pathway, NOD-like receptor signaling pathway, TNF signaling pathway, and VEGF signaling pathway (Figure 5A). Subsequent KEGG classification and pathway annotation analysis further categorized the enriched pathways into three major functional groups: human diseases (pathways in cancer, pertussis, lipid and atherosclerosis, inflammatory bowel disease, leishmaniasis, measles, non-alcoholic fatty liver disease, bladder cancer, malaria and alcoholic liver disease), organismal systems (immune system and endocrine system), and signal transduction (HIF-1 signaling pathway, TNF signaling pathway, PI3K-Akt signaling pathway, VEGF signaling pathway and NF-kappa B signaling pathway) (Figures 5B,C). Finally, a targets-pathways interaction network was constructed to visualize the associations between enriched pathways and key targets, with pathways shown in purple and targets in blue (Figure 5D).

### The evaluation of and toxicity

To ensure the therapeutic safety of key substrates, toxicity profiling was conducted—an essential step prior to clinical application. SwissADME analysis indicated that several compounds, including *5-(3,4-Dihydroxyphenyl)-4-hydroxypentanoic acid*, *secoisolariciresinol*, and *naringenin chalcone*, satisfied Lipinski’s rule of five, demonstrating favorable drug-likeness (Table 1). Results from ADMETlab further revealed that these gut microbiota metabolites exhibited low to moderate risk levels in terms of carcinogenicity, hERG inhibition, human hepatotoxicity (H-HT), drug-induced liver injury (DILI), and oral LD₅₀ (Tables 2, 3). These findings suggest that the selected substrates possess acceptable toxicity profiles and may have potential for clinical application, although further validation is required.

**Table 1:** The evaluation of toxicity on key metabolites.

Compound	MW	HBA	HBD	MLOGP	Lipinski violations	Bioavailability score	TPSA
5-(3,4-Dihydroxyphenyl)-4-hydroxypentanoic acid	226.23	5	4	0.55	0	0.56	97.99
Secoisolariciresinol	362.42	6	4	1.56	0	0.55	99.38
Naringenin chalcone	272.25	5	4	1.02	0	0.55	97.99
3-(3,4-Dihydroxyphenyl)-2-hydroxypropanoic acid	198.17	5	4	−0.04	0	0.56	97.99
1-O-Caffeoylglycerol	254.24	6	4	−0.07	0	0.55	107.22
Myristic acid	228.37	2	1	3.69	0	0.85	37.3
10-Keto-12Z-octadecenoic acid	296.44	3	1	3.59	0	0.85	54.37
Linoleic acid	280.45	2	1	3.59	0	0.85	37.3
10-Oxo-11-octadecenoic acid	296.44	3	1	3.59	0	0.85	54.37
2-Hydroxy-3-(4-hydroxyphenyl)propanoic acid	182.17	4	3	0.52	0	0.56	77.76

Lipinski’s rule of five: MW <500; HBA <10; HBD ≤5; MLOGP ≤4.15; Lipinski violations ≤1; bioavailability score >0.1; TPSA <140.

**Table 2:** The evaluation of toxicity on key metabolites.

Compound	hERG	H-HT	DILI	Carcinogenicity	LD50_oral
5-(3,4-Dihydroxyphenyl)-4-hydroxypentanoic acid	0.015	0.202	0.032	0.12	0
Secoisolariciresinol	0.132	0.253	0.208	0.089	0
Naringenin chalcone	0.141	0.076	0.77	0.524	0
3-(3,4-Dihydroxyphenyl)-2-hydroxypropanoic acid	0.02	0.252	0.377	0.029	0
1-O-Caffeoylglycerol	0.007	0.057	0.048	0.341	0
Myristic acid	0.04	0.03	0.042	0.078	0
10-Keto-12Z-octadecenoic acid	0.017	0.106	0.059	0.149	0
Linoleic acid	0.031	0.174	0.016	0.351	0
10-Oxo-11-octadecenoic acid	0.035	0.125	0.102	0.222	0
2-Hydroxy-3-(4-hydroxyphenyl)propanoic acid	0.029	0.14	0.281	0.031	0

**Table 3:** Explanation of parameter range for drug toxicity evaluation.

Parameter	Definition	Range and interpretation
Carcinogenicity	Potential risk of cancer induction caused by the compound	- <0.3: Low risk
		- 0.3–0.7: Moderate risk
		- >0.7: High risk
hERG Inhibition	Cardiotoxicity risk due to inhibition of the hERG potassium channel, which may cause QT prolongation and arrhythmias	- <0.1: Low risk
		- 0.1–0.3: Moderate risk
		- >0.3: High risk
H-HT	Human hepatotoxicity: likelihood of liver cell damage induced by the compound	- <0.2: Low risk
		- 0.2–0.5: Moderate risk
		- >0.5: High risk
DILI	Drug-induced liver injury: prediction of clinically relevant hepatotoxicity	- <0.1: Low risk
		- 0.1–0.3: Moderate risk
		- >0.3: High risk
LD50_oral	Oral median lethal dose: estimated dose causing death in 50% of rodents (unit: mg/kg)	- >5,000: Practically non-toxic (category 5)
		- 300–2,000: Moderately toxic (category 3–4)
		- <50: Highly toxic (category 1)

### The “M-S-M-T” network analysis

**Figure 6:**
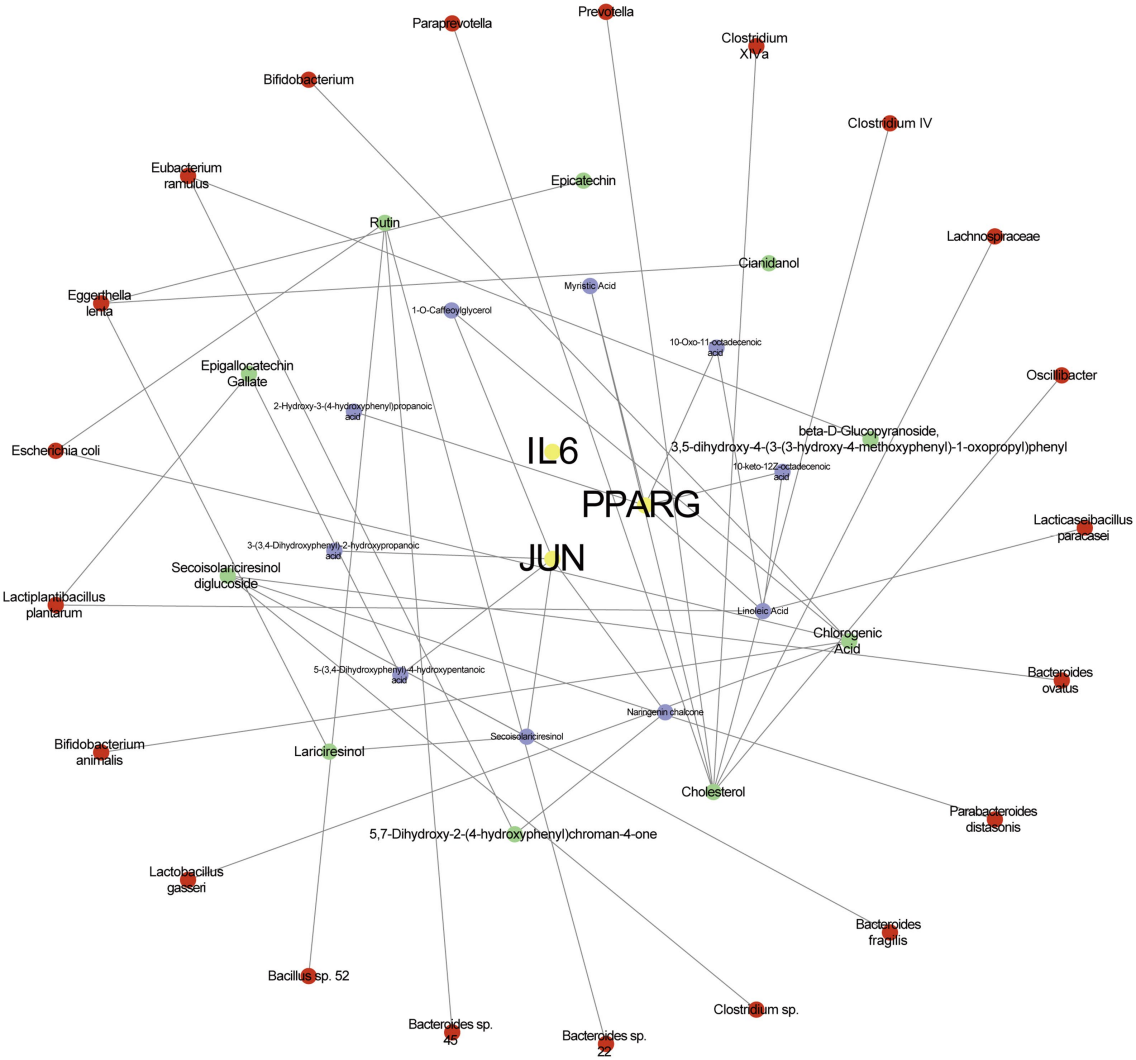
The network of microbiota-substrate-metabolites-targets. The yellow color represents targets, the green color represents metabolites, the purple color represents substrate and the red color represents gut microbiota.

Finally, we constructed a “Microbiota-Substrate-Metabolite-Target” (M-S-M-T) network to visualize the complex interactions among gut microbiota, their metabolic substrates, metabolites, and targets. This network revealed three hub genes—IL6, JUN, and PPARG—as well as their associations with 10 metabolites, 10 substrates, and 21 gut microbial taxa (Figure 6). In the network diagram, the yellow color represents targets, the green color represents metabolites, the purple color represents substrate and the red color represents gut microbiota. Notably, JUN exhibited the highest degree of connectivity with metabolites and substrates. All microbial species included in the network were clearly annotated and may serve as potential therapeutic targets for insulin resistance.

## Discussion

Insulin resistance (IR) is a common and complex metabolic disorder that has emerged as a major global health concern. It is characterized by diminished cellular sensitivity to insulin, leading to impaired glucose uptake and utilization, and consequently elevated blood glucose levels. Globally, the prevalence of IR continues to rise steadily; approximately 1 in 11 adults has diabetes, with type 2 diabetes mellitus (T2DM) accounting for 90% of the cases (Zheng et al., 2018). Alarmingly, IR is no longer confined to adults—it is increasingly observed in children and adolescents, compounding the long-term health burden (Jia et al., 2024). More importantly, IR not only contributes directly to the onset and progression of T2DM but also serves as a shared pathological basis for several metabolic disorders, including cardiovascular diseases, nonalcoholic fatty liver disease (NAFLD), and polycystic ovary syndrome (PCOS) (Lee et al., 2022). These complications markedly impair quality of life and impose significant economic burdens on healthcare systems. Therefore, the identification of effective therapeutic strategies to mitigate the impact of IR is of paramount importance.

Emerging evidence suggests that modulation of gut microbiota composition and abundance holds promise as a therapeutic approach for IR. This bidirectional regulation encompasses both the influence of host behaviors on the gut microbiota and the impact of microbial modulation on host metabolic parameters. For instance, a high-fiber diet can enhance the abundance of short-chain fatty acid (SCFA)-producing bacteroidales while reducing proinflammatory bacteria, thereby decreasing intestinal monosaccharide (e.g., fructose, galactose) accumulation and improving insulin sensitivity (Sonnenburg and Sonnenburg, 2014). Physical exercise promotes microbial diversity and has been shown to alleviate IR (Allen et al., 2018). Additionally, adequate sleep and stress management modulate gut microbiota-derived metabolites along the gut-brain axis, thereby enhancing insulin responsiveness (Zhao et al., 2022).

Conversely, targeted microbiota interventions can also improve metabolic outcomes. Supplementation with *Alistipes indistinctus* significantly reduces hepatic triglyceride accumulation and fasting glucose levels in IR mouse models (Semo et al., 2024). *Akkermansia muciniphila*, a mucin-degrading bacterium, enhances gut barrier integrity, reduces endotoxin translocation, and stimulates GLP-1 secretion, collectively contributing to improved insulin signaling (Depommier et al., 2019).

Network pharmacology, rooted in systems biology and multi-omics integration, facilitates the construction of drug-target-disease networks to elucidate the molecular mechanisms of therapeutic agents. Its ability to systematically predict drug targets and efficacy, identify novel target-pathway associations, and support precision medicine makes it an invaluable tool in drug discovery and disease modeling (Nogales et al., 2022). To uncover key targets and metabolites in IR treatment, we constructed a comprehensive “disease-gene-gut microbiota-metabolite” interaction network using data from public databases.

Protein–protein interaction (PPI) analysis identified IL6, JUN, and PPARG as hub genes in IR, each functioning through distinct yet interconnected mechanisms: IL6 mediates inflammation, JUN is involved in oxidative stress signaling, and PPARG regulates lipid metabolism. IL6, a prototypical proinflammatory cytokine, activates the JAK/STAT3 pathway, inducing SOCS3 expression in adipose and hepatic tissues. SOCS3 inhibits tyrosine phosphorylation of insulin receptor substrate-1 (IRS-1), thereby disrupting PI3K/Akt signaling and reducing insulin sensitivity (Senn et al., 2002). JUN (c-Jun), a component of the AP-1 transcription factor complex, is activated via the JNK pathway under oxidative stress or high-fat dietary conditions. It promotes inflammatory gene expression and lipogenesis while impairing GLUT4 translocation, thus exacerbating insulin resistance in adipose tissue and the liver (Hirosumi et al., 2002). PPARG plays a pivotal role in lipid metabolism by promoting adipocyte differentiation, enhancing adiponectin secretion, and reducing lipotoxicity in peripheral tissues. Moreover, PPARG suppresses NF-κB-mediated inflammation and improves glucose uptake in muscle and liver while indirectly modulating gut microbiota-derived metabolites to enhance insulin signaling (Ahmadian et al., 2013; Rangwala and Lazar, 2004). Collectively, these factors form an inflammation-metabolism-stress regulatory network, offering multiple points for therapeutic intervention in IR.

KEGG pathway enrichment analysis revealed that the IL-17 signaling pathway, Toll-like receptor (TLR) signaling pathway, and HIF-1 signaling pathway play crucial roles in IR regulation. The IL-17 pathway, activated by Th17 cell-derived IL-17A, stimulates the MAPK/NF-κB cascade, promoting the expression of proinflammatory cytokines (e.g., IL-6, TNF-α) in metabolic tissues. This leads to macrophage polarization toward the M1 phenotype and enhanced serine phosphorylation (but reduced tyrosine phosphorylation) of IRS-1, thereby inhibiting insulin signaling (Bapat et al., 2015; Zúñiga et al., 2010). The TLR pathway recognizes gut-derived endotoxins and free fatty acids, activating NF-κB and JNK via MyD88-dependent mechanisms. This not only triggers inflammatory responses and SOCS3 expression but also disrupts intestinal barrier integrity, exacerbating systemic inflammation and metabolic endotoxemia—conditions that are alleviated by TLR4 inhibition (Cani et al., 2007). HIF-1 signaling, induced by hypoxic adipose environments in obesity, upregulates glycolytic enzymes (e.g., LDHA) and angiogenic factors (e.g., VEGF), contributing to adipocyte hypertrophy and fibrosis. Simultaneously, HIF-1α promotes lipid synthesis by inhibiting mitochondrial oxidative phosphorylation, thereby aggravating lipotoxicity and IR (Halberg et al., 2009). These pathways converge into an interrelated network of inflammation, metabolism, and hypoxia, offering multidimensional therapeutic entry points.

Among the identified substrates, cholesterol, chlorogenic acid, and rutin emerged as key metabolites. Cholesterol modulates IR via liver X receptor (LXR)-mediated reverse cholesterol transport, enhancing ABCA1/ABCG1 expression to prevent macrophage lipid accumulation while suppressing SREBP-1c-driven lipogenesis. In contrast, cholesterol oxidation products exacerbate IR via ER stress and NF-κB activation, indicating that their inhibition may preserve β-cell function and reduce hepatic steatosis. Chlorogenic acid improves IR by activating the AMPK/PGC-1α axis, enhancing mitochondrial metabolism, suppressing gluconeogenic enzymes (PEPCK, G6Pase), and fortifying intestinal mucosal integrity, thereby reducing endotoxin leakage and systemic inflammation (Horton et al., 2002). In an *in vitro* study conducted by Onyango (2018), hepatocytes and adipocytes cultured in vitro were stimulated with cholesterol, resulting in the activation of downstream NF-κB and JNK pathways of the Toll-like receptor 4 (TLR4) signaling cascade. Concurrently, phosphorylation levels of IRS-1/Akt were reduced, and GLUT4 expression was downregulated, indicating that cholesterol induces insulin resistance through TLR4-mediated activation of multiple stress response pathways. Similarly, research by Radin et al. (2008) demonstrated that knockout of TLR4 or treatment with TLR4 inhibitors (e.g., TAK-242) in adipocytes significantly reduced the expression of cholesterol-induced proinflammatory cytokines and restored insulin signaling activity, suggesting a negative correlation between the TLR4 pathway and the development of insulin resistance.

In an *in vivo* animal study, Ye et al. (2021) investigated the effects of chlorogenic acid (CGA) on obesity and related metabolic endotoxemia, as well as its association with gut microbiota alterations and insulin regulation. Oral glucose tolerance tests (OGTT) and insulin tolerance tests (ITT) were performed, alongside measurements of inflammatory and gut barrier function markers. The composition of the gut microbiota was analyzed using 16S rRNA high-throughput sequencing. The results showed that CGA suppressed high-fat diet (HFD)-induced weight gain and fat accumulation in mice, independent of food intake, indicating improved glucose homeostasis and reduced insulin resistance. Moreover, CGA decreased plasma lipopolysaccharide (LPS) levels and inhibited the expression of TLR-4, TNF-α, IL-1β, and MCP-1 in the liver and epididymal adipose tissue, thereby alleviating low-grade inflammation and enhancing insulin sensitivity. In addition, CGA increased the abundance of short-chain fatty acid (SCFA)-producing bacteria in the gut, improved gut barrier function, and reduced LPS translocation into the bloodstream. Fecal microbiota transplantation experiments further demonstrated that mice receiving microbiota from the HCGA group exhibited reduced body weight and fat mass, along with improved insulin sensitivity and glucose tolerance.

Zhang’s research team identified cholesterol, chlorogenic acid, and rutin as key metabolites through metabolomic screening and analyzed their co-occurrence with 21 gut microbial taxa using the GutMDisorder database. The results revealed that in HFD-fed mice, rutin increased the abundance of *Akkermansia muciniphila*, activated the aryl hydrocarbon receptor (AHR) signaling pathway, and promoted the production of the tryptophan-derived metabolite indole-3-acetic acid (IAA). These effects collectively suppressed the hepatic TLR4/NF-κB inflammatory pathway (targeting IL-6) and downregulated lipid synthesis-related genes (Scd-1, Fasn, Acaca). The findings suggest that rutin alleviates IR-related metabolic disturbances through a “microbiota-metabolite-host” axis (Ye et al., 2021).

Furthermore, *Eggerthella lenta* was identified as a key microbiota in the M-S-M-T network. This species transforms host-derived bile acids into secondary bile acids, which activate intestinal FXR and TGR5 receptors to suppress hepatic gluconeogenesis, promote GLP-1 secretion, and enhance β-cell function. Its metabolites also inhibit the TLR4/NF-κB pathway, reduce adipose macrophage infiltration, and lower proinflammatory cytokine levels, ultimately restoring insulin sensitivity (Paik et al., 2022).

Despite the promising role of the gut microbiota in the treatment of insulin resistance (IR) revealed in this study, several limitations should be acknowledged. First, the gut microbiota data utilized in this work were derived from the gutMGene database, which is primarily based on fecal samples. Although such data provide a valuable reference, they may not fully reflect the actual composition of intestinal microbiota compared to biopsy-derived samples, and are subject to considerable inter-individual variability. As a result, the constructed network may carry certain biases, and the reliability of the microbial data should be further validated using experimental approaches such as fecal microbiota transplantation (FMT), 16S rRNA sequencing, and metagenomic analysis.

Moreover, this study mainly focused on IR itself, whereas the roles of gut microbiota and their metabolites in IR-related complications, such as hypertension and hyperlipidemia, remain underexplored. Future investigations should place greater emphasis on these aspects.

To enhance the robustness of the findings, future studies should integrate network pharmacology with multi-omics approaches—including metagenomics and metabolomics—to establish a more comprehensive regulatory network. Furthermore, systematic validation through animal experiments and clinical trials is essential to clarify the mechanisms by which gut microbiota contribute to the regulation of IR and its associated metabolic disorders.

## Conclusion

In this study, we employed network pharmacology approaches to elucidate the regulatory mechanisms by which the gut microbiota contributes to the pathogenesis of insulin resistance (IR). Three hub genes—IL6, JUN, and PPARG—were ultimately identified as potential therapeutic targets for IR. These genes correspond to key pathological aspects of IR: inflammatory regulation (IL6), stress-related signaling (JUN), and lipid metabolic balance (PPARG), providing distinct avenues for targeted intervention.

By constructing the microbiota-substrate-metabolite-target (M-S-M-T) network, we revealed that these three hub genes are closely associated with 10 gut microbiota metabolites, 10 microbial substrates, and 21 Gut microbiota. Among these, cholesterol, chlorogenic acid, and rutin emerged as key compounds that improve IR and its complications through mechanisms including lipid metabolism regulation, mitigation of oxidative stress, and modulation of microbiota-host interactions. For example, inhibiting intestinal cholesterol absorption has been shown to alleviate hepatic steatosis and contribute to the amelioration of IR, highlighting a mechanistic axis of therapeutic relevance.

Additionally, KEGG enrichment analysis identified six major signaling pathways implicated in IR pathophysiology: the IL-17 signaling pathway, Toll-like receptor (TLR) signaling pathway, HIF-1 signaling pathway, NOD-like receptor signaling pathway, TNF signaling pathway, and VEGF signaling pathway. Notably, IL-17, TLR, and HIF-1 signaling pathways play pivotal roles in reshaping the inflammatory microenvironment, responding to metabolic endotoxemia, and mediating hypoxia-induced metabolic imbalance, respectively. These three pathways collectively form an interrelated inflammation–metabolism–hypoxia regulatory network. Targeting key nodes within this network—such as the IL-17 receptor, TLR4, or HIF-1α—may offer multi-dimensional therapeutic strategies for IR and its associated complications.

## Data Availability

The original contributions presented in the study are included in the article/supplementary material, further inquiries can be directed to the corresponding author.

## Footnotes

^1^ http://bio-computing.hrbmu.edu.cn/gutmgene/#/Resource

^2^ https://pubchem.ncbi.nlm.nih.gov/

^3^ https://www.genecards.org/

^4^ https://disgenet.com/

^5^ https://www.omim.org/

^6^ https://string-db.org

^7^ https://david.ncifcrf.gov/tools.jsp

^8^ http://www.swissadme.ch/index.php

^9^ https://admetmesh.scbdd.com/
